# Materials and Design Approaches for a Fully Bioresorbable, Electrically Conductive and Mechanically Compliant Cardiac Patch Technology

**DOI:** 10.1002/advs.202303429

**Published:** 2023-07-30

**Authors:** Hanjun Ryu, Xinlong Wang, Zhaoqian Xie, Jihye Kim, Yugang Liu, Wubin Bai, Zhen Song, Joseph W. Song, Zichen Zhao, Joohee Kim, Quansan Yang, Janice Jie Xie, Rebecca Keate, Huifeng Wang, Yonggang Huang, Igor R. Efimov, Guillermo Antonio Ameer, John A. Rogers

**Affiliations:** ^1^ Department of Advanced Materials Engineering Chung‐Ang University Anseong 17546 Republic of Korea; ^2^ Department of Intelligence Energy and Industry Chung‐Ang University Seoul 06974 Republic of Korea; ^3^ Department of Biomedical Engineering Northwestern University Evanston IL 60208 USA; ^4^ Center for Advanced Regenerative Engineering Northwestern University Evanston IL 60208 USA; ^5^ State Key Laboratory of Structural Analysis Optimization and CAE Software for Industrial Equipment Dalian University of Technology Dalian 116024 P. R. China; ^6^ Department of Engineering Mechanics Dalian University of Technology Dalian 116024 P. R. China; ^7^ DUT‐BSU Joint Institute Dalian University of Technology Dalian 116024 P. R. China; ^8^ Querrey Simpson Institute for Bioelectronics Northwestern University Evanston IL 60208 USA; ^9^ Department of Applied Physical Sciences University of North Carolina at Chapel Hill Chapel Hill NC 27599 USA; ^10^ Departments of Civil and Environmental Engineering, Mechanical Engineering, and Materials Science and Engineering Center for Bio‐integrated Electronics Northwestern University Evanston IL 60208 USA; ^11^ Department of Biomedical Engineering Northwestern University Chicago IL 60611 USA; ^12^ Department of Medicine Northwestern University Chicago IL 60611 USA; ^13^ Department of Surgery Feinberg School of Medicine Northwestern University Chicago IL 60611 USA; ^14^ Chemistry of Life Processes Institute Northwestern University Evanston IL 60208 USA; ^15^ International Institute for Nanotechnology Northwestern University Evanston IL 60208 USA; ^16^ Simpson Querrey Institute for Bionanotechnology Evanston IL 60208 USA; ^17^ Department of Mechanical Engineering Northwestern University Evanston IL 60208 USA; ^18^ Department of Materials Science and Engineering Northwestern University Evanston IL 60208 USA; ^19^ Department of Neurological Surgery Feinberg School of Medicine Northwestern University Chicago IL 60611 USA

**Keywords:** bioresorbable materials, cardiac patch, heterogeneous integration, myocardial infraction

## Abstract

Myocardial infarction (MI) is one of the leading causes of death and disability. Recently developed cardiac patches provide mechanical support and additional conductive paths to promote electrical signal propagation in the MI area to synchronize cardiac excitation and contraction. Cardiac patches based on conductive polymers offer attractive features; however, the modest levels of elasticity and high impedance interfaces limit their mechanical and electrical performance. These structures also operate as permanent implants, even in cases where their utility is limited to the healing period of tissue damaged by the MI. The work presented here introduces a highly conductive cardiac patch that combines bioresorbable metals and polymers together in a hybrid material structure configured in a thin serpentine geometry that yields elastic mechanical properties. Finite element analysis guides optimized choices of layouts in these systems. Regular and synchronous contraction of human induced pluripotent stem cell‐derived cardiomyocytes on the cardiac patch and ex vivo studies offer insights into the essential properties and the bio‐interface. These results provide additional options in the design of cardiac patches to treat MI and other cardiac disorders.

## Graphical abstract

A bioresorbable, highly conductive, and elastic cardiac patch (BCEP) designed as a heterogeneous material structure for myocardial infarction treatment. Finite element analysis defines geometries of filamentary serpentine mesh architectures in a bioresorbable metal for optimized elastic properties when integrated with a bioresorbable elastomer. Human‐induced pluripotent‐stem‐cell‐derived cardiomyocytes grown on the BCEP demonstrate regular and synchronous contractions. Ex vivo studies highlight the ability of the BECP to form electroconductive pathways for transmitting the bioelectrical signals.

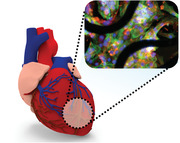

## 1 Introduction

Cardiovascular disease is one of the leading causes of morbidity and mortality, expected to affect approximately 23.6 million people globally by 2030.^[^ ^1^, ^2^ ^]^ Cardiovascular disease can often lead to MI, the end result of the obstruction of coronary arteries and associated restriction in blood flow to cardiomyocytes. The result leads to the replacement of parts of the myocardium with scar tissue,^[^ ^3^ ^]^ typically within the wall of the left ventricle (LV), that inhibits propagation of electrical signals and desynchronizes LV contraction, eventually leading to heart failure.^[^ ^4^ ^]^ Heart transplantation can be an effective treatment, but limits on suitable donors and the potential for immune rejection remain major challenges.^[^ ^5^ ^]^ The current approaches to treat MI (e.g., stem‐cell transplantation,^[^ ^6^, ^7^ ^]^ biomimetic scaffolds,^[^ ^8^ ^]^ cardiac patch,^[^ ^9^, ^10^, ^11^, ^12^ ^]^ and pharmacotherapy^[^ ^13^, ^14^ ^]^) aim to prevent the formation of scar tissues to recover their native electrical and mechanical properties. Experimental trials of stem‐cell transplantation show promising outcomes in preclinical models;^[^ ^15^ ^]^ however, inconsistencies with the experiments, limited cell viability after transplantation, and functional integration into the host myocardium must be addressed through further work on this approach.^[^ ^16^ ^]^

Cardiac patches, which typically consist of electroconductive materials, elastic substrates, and/or cardiac tissues with properties similar to those of the normal myocardium, represent an alternative form of treatment. These patches improve the contractile function as well as synchronize abnormal areas with slow electrical conduction in the damaged heart to prevent life‐threatening reentrant arrhythmia and to thereby restore normal electrical conduction.^[^ ^17^ ^]^ Electroconductive materials, such as gold (Au), silicon (Si), carbon‐based materials (e.g., graphene, carbon nanotubes, carbon dots), and conductive polymers (e.g., polypyrrole (PPy), polyaniline (PANI)), integrated with substrates such as decellularized bovine pericardium tissue, chitosan, polylactic acid (PLA), collagen, and gelatin can be engineered with the appropriate levels of electroconductivity, mechanical stiffness, and elongation for these applications.^[^ ^18^, ^19^ ^]^ Cytotoxicity of the electroconductive materials is an important factor in determining the concentration of the material to be used for cardiac patch fabrication. Currently, available cardiac patches on the market are nonbiodegradable polytetrafluoroethylene (PTFE) and biodegradable bovine pericardium, which usually function as mechanical support. As myocardium remodeling progresses in the first two months following MI to form scar tissue that preserves LV wall integrity, the development of a bioresorbable cardiac patch that can temporarily provide both mechanical and electrical support to reduce the remodeling process and preserve cardiac function will benefit the patients.^[^ ^20^ ^]^ In addition, the bioresorbable cardiac patch can also facilitate the integration with host tissue by reducing the harshness of the microenvironment around it.^[^ ^21^, ^22^ ^]^ The transient nature of the bioresorbable devices offers additional benefits due to significant differences in cardiac electro‐mechanics of the healed post‐MI heart versus the heart during the acute MI healing state, characterized by post‐repolarization refractoriness, ectopic activity, and reduced mechanical activity of the ischemic myocardium.

Herein, we introduce a bioresorbable, highly conductive, and elastic cardiac patch (BCEP) that provides electrical conduction pathways, mechanical support, and biocompatible surfaces for the growth of cardiac cells. The BCEP consists of a highly conductive bioresorbable metal (molybdenum for the cases presented here due to its slow dissolution rate, but with magnesium, zinc, and iron as alternatives^[^ ^23^ ^]^) mesh structure formed in an optimized filamentary serpentine geometry, a poly(1,8‐octamethylene‐citrate‐co‐octanol) (POCO) elastic polymer substrate, and a bioadhesive polyethylene glycol lactide acid diacrylate (PEG‐LA‐DA) hydrogel to promote adhesion to the tissue.^[^ ^24^ ^]^ The serpentine mesh design ensures sufficient stretchability, with high area coverage of metal, where bioresorption occurs via hydrolysis in surrounding biofluids after the treatment period, to eliminate the need for post‐surgical retrieval. Cytotoxicity tests and active cardiac cells formed on the BCEP demonstrate its potential as a bioresorbable therapeutic solution. Ex vivo studies illustrate the detection of electrocardiogram (ECG) signals and electroconductive pathways. This technology may offer a simple and effective option in the treatment of certain classes of cardiac disorders.

## 2 Results and Discussion

**Figure 1:**
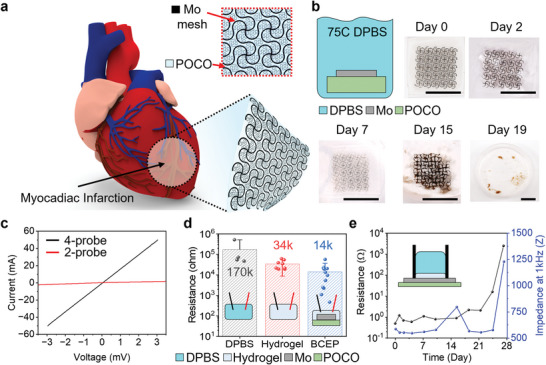
a) Schematic illustration of a BCEP mounted on the epicardial surface. The inset magnified image highlights the hybrid materials structure of the BCEP. b) Optical images of accelerated dissolution of the BCEP when immersed in DPBS (pH = 7.4) at 75 °C; scale bar: 1 cm. c) Four‐probe current and resistance versus voltage measurements from a sample of BCEP. d) Average resistance of DPBS (pH = 7.4), bioadhesive hydrogel, and BCEP. The results are shown as mean ± SD. A value of *p* ≤ 0.05 is considered to indicate a significant difference. e) Changes in resistance and impedance at 1 kHz as a function of time of immersion in DPBS (pH 7.4) at 50 °C.

MI can result from occlusion of the left anterior descending artery, resulting in damage to the LV wall. **Figure** **1**a shows a schematic illustration of a BCEP applied to a region of MI on the LV wall. A bioadhesive hydrogel (PEG‐LA‐DA, alginate, and chitosan mixture; Young's modulus ≈30 kPa, elongation ≈10 times, ionic conductivity ≈0.5 S m^−1^, impedance ≈8 kΩ at 1 kHz in 0.1 M phosphate‐buffered saline (PBS) at room temperature^[^ ^24^ ^]^) bonds the BCEP to the surface of the heart. The image on the top right side shows the Mo mesh structure on a POCO polymer substrate (120 µm thick), as the basis for a bendable, stretchable, and highly conductive framework. The major serpentine trace of this mesh (size: 1.2 × 1.2 cm) has a width of 100 µm and thickness of 15 µm, as the primary pathway for electrical conduction. The additional traces (widths of 20 µm and thicknesses of 15 µm) adopt the geometry of a fractal‐inspired minor Greek cross, to increase the coverage of the conductive area by ≈30%. The minimum and maximum distances between adjacent traces are 80 and 140 µm, respectively, (Figure S1, Supporting Information). Since the average human cardiomyocyte is approximately 80–120 µm in length and 20–30 µm in width,^[^ ^25^, ^26^ ^]^ this mesh provides direct electrical interfaces to nearly all cells that exist near the surface of the cardiac tissue. The flexible and stretchable POCO substrate (thickness of 120 µm) provides an elastic support for the BCEP, with an abundance of surface carboxyl groups (‐COOH) that can react with amine groups in the bioadhesive hydrogel.^[^ ^27^ ^]^

Figure 1b highlights the experimental setup for evaluating the processes of dissolution of the BCEP, as well as photographs at various time points after immersion in Dulbecco's PBS (DPBS; pH 7.4) at 75 °C, as the basis for accelerated testing (≈16 times relative to body temperature).^[^ ^28^ ^]^ The Greek cross traces dissolve after 2 days, and most of the mesh structure breaks into fragments after 7 days. The POCO substrate loses its shape after 15 days, and most of the BCEP dissolves after 19 days. Acceleration test results indicate that the Greek cross traces dissolve in a month and that the conductivity of BCEP disappears in a few months. The BCEP has a low DC resistance (∼0.2 Ω/sq, see Figure 1c, Figure S1, Supporting Information). The impedance at the interface with the DPBS is 25 Ω at 1 kHz frequency (see Figure S2, Supporting Information). The average resistance of the BCEP when fully covered by the hydrogel is lower than that of the DPBS and the hydrogel (*p* < 0.05, Figure 1d). Figure 1e summarizes tests of electrical degradation of the BCEP when covered by bioadhesive hydrogel and immersed in DPBS (pH 7.4) at 50 °C. The conductivity and interface impedance at 1 kHz frequency remain unchanged over 3 weeks. Due to pitting corrosion at the interface of the Mo mesh and the PBS container, accelerated corrosion‐induced fracture occurs and leads to a loss of electroconductivity after 3.5 weeks.

**Figure 2:**
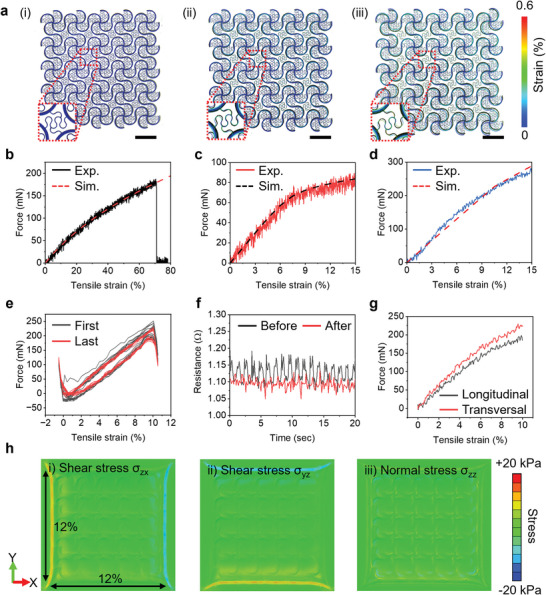
a) Optical images and overlaid simulation results of BCEPs stretched by (i) 0%, (ii) 6%, and (iii) 12%; scale bar: 2 mm. Force as a function of uniaxial tensile strain for b) POCO substrate, c) Mo mesh, and d) BCEP. e) Force as a function of uniaxial tensile strain of a BCEP before and after 10 000 cycles of stretch–release to 10% strain. f) Changes in resistance of a BCEP as a function of time during stretching (to 10% strain) and release before (black) and after (red) 10 000 cycles. g) Force as a function of uniaxial tensile strain for a BCEP coated with bioadhesive hydrogel along the longitudinal and transversal axes. h) Simulation results for the distribution of shear and normal stresses at the interface between the BCEP and underlying cardiac tissue during biaxial stretching.

**Figure** **2**a shows overlaid optical images and finite element analysis (FEA) results for the Mo mesh at various levels of biaxial strain. The equivalent strain in Mo remains below 0.6% for 12% biaxial stretching, and 90° twisting (Figure S3b, Supporting Information). The maximum equivalent strain in the Mo mesh layer is less than ≈0.3% for a bending radius of ≈3.8 mm (Figure S3a, Supporting Information). The strains are significantly less than thefracture strain (5%) of Mo for above‐critical deformations. These results highlight the robust, yet stretchable properties that can operate under realistic physiological loads. These designs also lead to a low effective modulus, as a soft interface that imposes minimal mechanical constraints on the natural movements of the heart (e.g., ≈13% circumferential and ≈10% longitudinal strain changes, and ≈10% volume changes^[^ ^29^ ^]^). The bulk modulus and tangential shear modulus of the post‐mortem human left ventricle are 0.25 GPa and 60–148 kPa, respectively.^[^ ^25^ ^]^ Figure 2b–d shows the tensile strain versus force curves of the POCO polymer substrate, Mo mesh, and BCEP, respectively. Uniaxial stretching of the POCO polymer shows elastic behaviors for up to 70% stretching (Figure 2b), but the Mo mesh shows a linear elastic deformation of ≈5% under a tensile stress of ≈30 mN (Figure 2c). Because the POCO substrate mainly serves as a soft and elastic bioresorbable support layer, the BCEP facilitates ≈15% linear elastic deformation corresponding to stretching of the cardiac surface, which can reach ≈13%.^[^ ^30^ ^]^ Tests under stretch–release cycles to strains of 10% at 1 Hz indicate electrical and physical stability through 10 000 cycles (Figure S4, Supporting Information). Figure 2e shows the tensile strain versus stress response before and after the 10000 test cycles, thus demonstrating that the elasticity of the BCEP remains unchanged. After this testing, the final resistance of the mesh is within ≈0.05 Ω of the initial resistance, ≈1.1 Ω (Figure 2f). Figure S5, Supporting Information presents the results of measurements of the mechanical properties of the bioadhesive hydrogel (thickness of 150–200 µm), as its role as a mechanical buffer layer to eliminate the possibility for damage to the cardiac tissue induced by the filamentary structures in the Mo mesh. This hydrogel does not significantly alter the elastic behaviors of the BCEP. Specifically, the BCEP with hydrogel has an elastic modulus of 235 ± 50 kPa along both the longitudinal and transversal axes, consistent with the radially symmetric structure of the Mo mesh (Figure 2g). Simulation results for the distribution of shear and normal stresses at the interface between the BCEP and underlying cardiac tissue during 12% biaxial stretching of the BCEP confirm that most of the interfacial stresses are significantly less than ∼20 kPa, the threshold of skin sensation^[^ ^31^ ^]^ (Figure 2h). The BCEP was applied on the heart of a Sprague Dawley (SD) rat, and no obvious interruption on heart contraction was observed indicating the BCEP is mechanically matched with the heart tissue (Video S1, Supporting Information).

**Figure 3:**
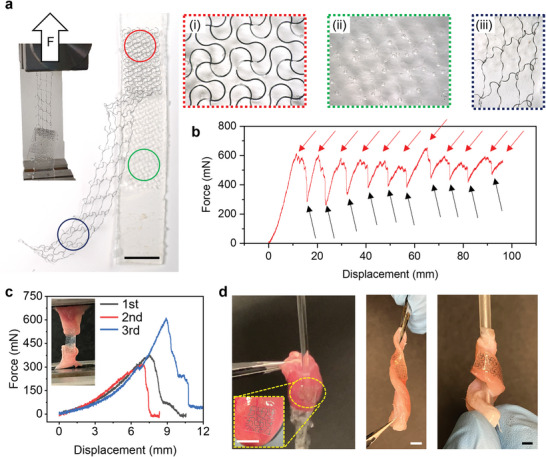
a) Optical images of the adhesion test setup and results. The inset frames show magnified optical images of the (i) Mo mesh adhered to the POCO (red box), (ii) POCO substrate after peeling of the Mo mesh (green box), and (iii) stretched Mo mesh after peeling from the POCO (blue box); scale bar: 1 cm. b) Applied force as a function of tensile displacement before and during peeling of the Mo mesh from the POCO. The black arrows correspond to times during elongation of the Mo serpentine structures in the vicinity of the POCO after peeling. The red arrows indicate the initiation of detachment of the Mo serpentines from the POCO. c) Force‐displacement test results of tissue‐BCEP. d) Examples of ex vivo mechanical stress and deformation tests.

The mesh remains bonded to the POCO throughout these tests. The strong adhesion follows mainly from mechanical interlocking. A long rectangular Mo mesh (1 cm width and 6 cm length) attached to a POCO substrate (1.2 cm width and 7 cm length) with ≈1 cm of the Mo mesh exposed at the end facilitates peel tests (**Figure** **3**a). The inset images highlight a bonded region of the sample (red box), a region of the POCO where the mesh has peeled away (green box), and a stretched region of the Mo mesh that has peeled away (blue box). Figure 3b shows a plot of applied force as a function of peel distance. The minimum peeling force along the longitudinal axis of the Mo mesh is ≈0.2 N (average initial peel force ≈0.4 N), and the maximum peeling force along the transversal axis of the Mo mesh is ≈0.6 N. The stepwise shape of this curve follows from the repeated peeling away of filaments in the serpentine. Figure S6, Supporting Information shows similar results obtained from a sample after soaking in DPBS for ≈24 h. The peeling force between the Mo mesh and swollen POCO substrate decreases to ≈0.3 N regardless of the longitudinal or transversal axis. Figure 3c demonstrates adherence between the BCEP and tissues. Three different samples indicate that the peeling force between the BCEP and tissue is ≈0.3N. Due to strong adhesion between the BCEP and tissues and the elastic properties of the system, mechanical stresses and deformations do not compromise the integrity of the BCEP (Figure 3d). Water rinse tests confirm that the bioadhesive hydrogel facilitates strong adhesion, to endure twisting deformations without failure.

**Figure 4:**
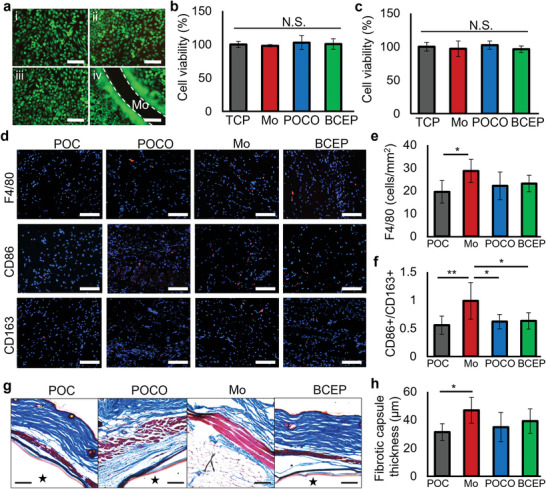
a) Live (green)/dead (red) staining of L929 fibroblasts on (i) TCP control, (ii) Mo mesh, (iii) POCO, and (iv) BCEP samples after 3 days of culture; scale bar: 100 µm. Cell viability tested by alamarblue assay using b) L929 fibroblasts and c) hiPSC‐CMs; N.S. represents no significant difference, *n* = 3. d) Immunofluorescent images of macrophage markers (F4/80, CD86 and CD163) in animals implanted with POC, POCO, Mo, and BCEP after 3 days; scale bar: 100 µm. Quantification of e) F4/80 and f) ratio of CD86+/CD163+ cells for inflammation evaluation of various implants. * *p* < 0.05, ** *p* < 0.01, *n* = 6 animals. g) Masson's trichrome staining shows the foreign body response to the implants, ★ indicates the implants; scale bar: 100 µm. h) The thickness of the fibrotic capsule around the implants after 4 weeks. * *p* < 0.05, *n* = 6 animals.

To evaluate the cytotoxicity of the patches, L929 fibroblasts were seeded onto (i) plastic substrate for the tissue culture (TCP control), (ii) Mo mesh, (iii) POCO, and (iv) BCEP sample according to ISO 10993–5:2009 standard (in vitro cytotoxicity evaluation of medical device). Live/dead staining after 3 days of culture serves as the basis for evaluating the cytotoxicity of each sample (**Figure** **4**a). According to the staining, most of the cells are alive in all samples. Quantification of cell viability using an alamarblue assay confirm that each component of the BCEP is not toxic (Figure 4b). In addition, the viability of human induced pluripotent stem cell‐derived cardiomyocytes (hiPSC‐CMs) was also quantified on various patches. The patches do not significantly influence hiPSC‐CMs viability, indicating the BCEP is biocompatible to cardiomyocytes (Figure 4c). The immune responses to the biomaterials were evaluated via subcutaneous implantation of the patches in C57BL/6J mice. A POC (poly(octanediol‐co‐citrate)) patch was implanted as a control, as previously proven to be biocompatible and used for medical device fabrication.^[^ ^32^ ^]^ 3 days postimplantation, the samples were collected and stained with macrophage markers including F4/80 (pan macrophage marker), CD86 (M1 macrophage marker), and CD163 (M2 macrophage marker) (Figure 4d). Quantification analysis show a higher F4/80 positive cell density and CD86+/CD163+ cell ratio with the Mo implant. However, the POCO and BCEP do not indicate any significant change in macrophage marker expression compared to POC, consistent with their biocompatibility (Figure 4e,f). This result is attributed to the intrinsic anti‐inflammation property of citrate‐based polymers.^[^ ^33^ ^]^ Masson's trichrome staining was performed at 4 weeks post‐implantation to evaluate the foreign body response. The fibrotic capsule was observed around the implant (Figure 4g). In accordance with the inflammation results, the combination of POCO with Mo (to fabricate the BCEP patch) significantly reduced the thickness of the fibrotic capsule (Figure 4h). The thicknesses of fibrotic capsules in all groups are below 50 µm, comparable to that for biodegradable polymers such as poly(glycerol sebacate) (PGS) and poly(L‐lactide‐co‐glycolide) (PLGA).^[^ ^34^ ^]^

**Figure 5:**
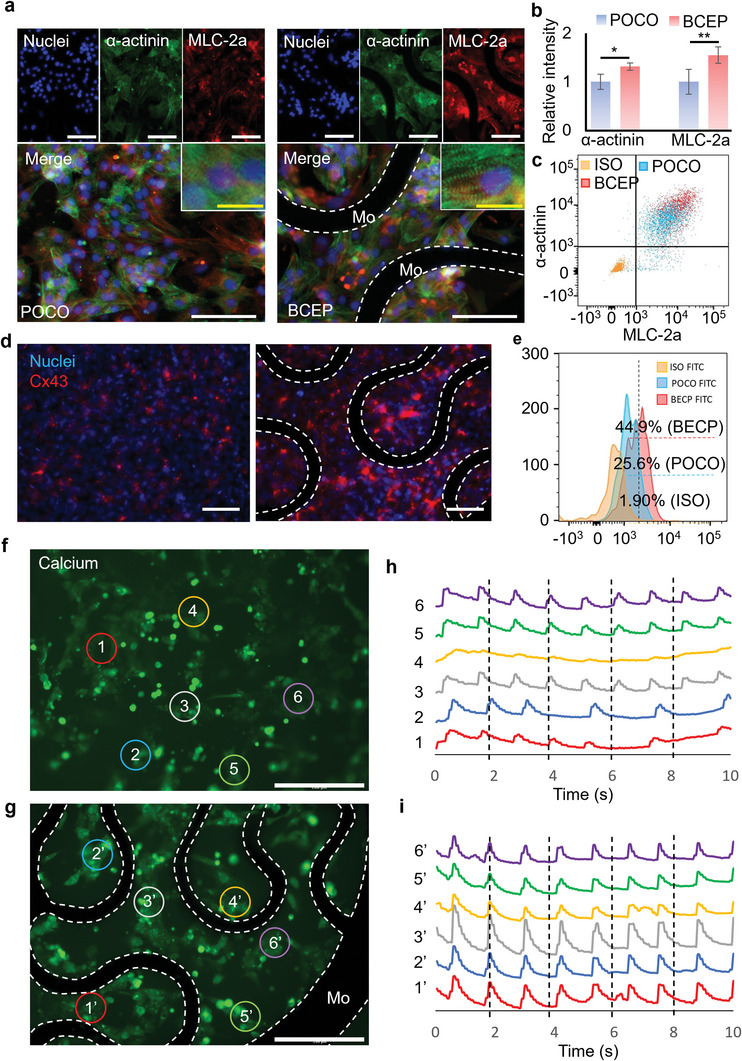
a) Representative immunofluorescent images of hiPSC‐CMs on POCO and BCEP; cell nuclei (blue) and specific markers of cardiomyocytes, including α‐actinin (green) and MLC‐2a (red) were visualized; images captured with high magnification show clear sarcomeric striations; white scale bar: 100 µm, yellow scale bar: 20 µm. b) The relative immunofluorescence intensities of α‐actinin and MLC‐2a, respectively; the results are normalized to those obtained with POCO, *n* = 6. c) Representative flow cytometric analysis diagram of hiPSC‐CMs probed for α‐actinin and MLC‐2a, respectively. d) Representative immunofluorescence images of hiPSC‐CMs probed for cell nuclei (blue) and Cx43 (red) on POCO and BCEP. e) Representative histogram of the flow cytometry analysis of the hiPSC‐CMs probed for Cx43 on POCO and BCEP. f) and g) are fluorescent images indicating Ca^2+^ flow of hiPSC‐CMs on POCO and BCEP; six regions of interest (ROIs) are randomly selected as shown to evaluate the cell contraction behaviors; white scale bar: 200 µm. The dashed lines in all the above images indicate the Mo serpentine structure in the BCEP. h) and (i) are traces of spontaneous contraction of six randomly selected regions on the POCO and BCEP.

Further evaluations of the influence of the BCEP on cardiomyocyte function use hiPSC‐CMs seeded onto the BCEP. A non‐conductive POCO patch represents the control. The hiPSC‐CMs attach and spread on the POCO and BCEP with a 0.1% gelatin coating. Most of the cells show positive staining for both α‐actinin and myosin light chain 2a (MLC2a), as typical cardiomyocyte markers, on the POCO and BCEP (**Figure** **5**a). The expressions of α‐actinin and MLC2a on the BCEP increase as the relative staining intensity increases (Figure 5b), suggesting that the addition of the Mo conductive path might promote maturation of the hiPSC‐CMs during in vitro culture. Flow cytometry analysis confirmed an increase of α‐actinin+/MLC2a+ cells from 86.6% to 97.5% (Figure 5c). Sarcomeric striations form on both surfaces, but the sarcomere length increased on BCEP compared to POCO indicating more structural maturation of the cells (Figure S7, Supporting Information). The gap junction connexin 43 (Cx43) was probed to evaluate cell‐cell communication, which is critical for the propagation of electrical depolarization in cardiomyocytes (Figure 5d). Enhanced expression of Cx43 was observed on conductive BCEP when compared to nonconductive POCO. Quantification with flow cytometry confirmed that the Cx43 positive cells increased from 25.6% to 44.9% with incubation on BCEP when compared to POCO (Figure 5e).

Besides maturation, the BCEP is expected to enhance the conduction of electrical impulses by providing conductive pathways that can facilitate synchronized cell contractions. To assess this function, treatment of hiPSC‐CMs with 0.25% trypsin‐EDTA for 5 min partially dissociates the cells, to allow subsequent seeding onto the POCO and BCEP. Isolated cell clusters that are not continuously connected to each other appear on both surfaces (Figure S8, Supporting Information). Cytoplasmic Ca^2+^ influx can be visualized using fluo‐4 AM to investigate the cell contraction behaviors. The hiPSC‐CMs show spontaneous contraction on both POCO and BCEP (Videos S2 and S3, Supporting Information). However, analysis of six randomly selected cell clusters on the POCO patch indicate irregular contraction with asynchronous rhythms, suggesting arrhythmia (Figure 5f,g). In contrast, the randomly selected cell clusters on the BCEP show regular and synchronous contractions (Figure 5h,i). Analysis of beating frequency suggest no significant difference of cell clusters on POCO and BCEP (Figure S9, Supporting Information). Variations in the beating frequency on POCO are however, much higher than on BCEP. This result indicates that the conductive pathways provided by the BCEP facilitate electrical connections among the cell clusters, thus ensuring synchronized contractions of the hiPSC‐CMs.

**Figure 6:**
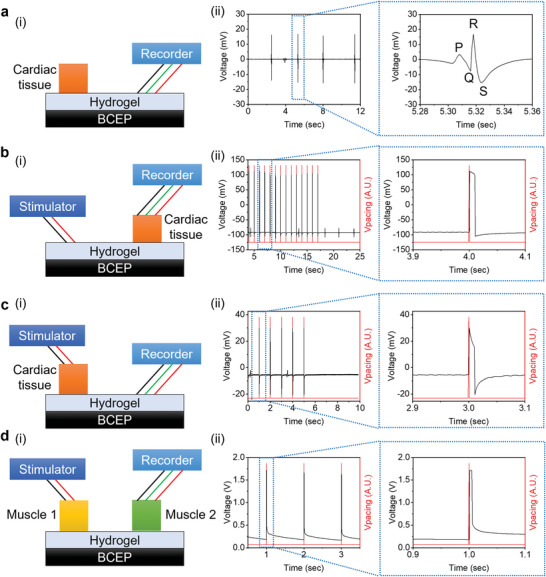
Evaluation of the electrical conduction properties of the BCEP under various experimental conditions: a) in situ ECG measurement through the BCEP, b) stimulation of cardiac tissue through the BCEP, c) detection of stimulating signals on the cardiac tissue through the BCEP, and d) stimulation of muscle tissue 1 with detection of the signal at muscle tissue 2 to demonstrate the MI model, where (i) is the schematic illustration of the experimental setup and (ii) shows the experimental results.

Further investigation of the influence of the BCEP on electrical signal propagation at the tissue level rely on an *ex vivo* experiment using LV myocardium and biceps femoris tissues collected from an adult rat. The schematic illustration in **Figure** **6**a(i) presents the experimental setup for measuring ECG data from the harvested cardiac tissue. The animal tissues adhere to the bioadhesive‐hydrogel‐coated BCEP via peptide bonds.^[^ ^24^ ^]^ A first test investigates whether the ECG signal from the cardiac tissue propagates through the Mo mesh. The results show a clear QRS waveform, confirming the negligible interfacial impedance and low resistance of the BCEP and the ability to increase the conduction velocity across the damaged electrical pathway (i.e., damaged area in Figure 6a(ii)). Figure 6b depicts the process of electrical stimulation to pace the cardiac tissue across the BCEP conducting path over 1 cm. Upon application of a pulsed DC waveform (1 V for 10 ms) to the BCEP, the cardiac tissue responds within 1 ms, which is faster than the longitudinal conduction velocity of a normal rat heart (maximum 80 cm s^−1^).^[^ ^35^ ^]^ Similarly, the application of a pulsed DC waveform (1 V for 10 ms) to the cardiac tissue registers a signal in the BCEP within 1 ms (Figure 6c). The capacitance between the two stimulation electrodes on the cardiac tissue yields a capacitive voltage response during stimulation. Between the electrostimulation intervals, natural ECG signals also appear in the recordings. An assay to test the bioconductivity between isolated muscle tissues serves as a mimic of the MI cardiac model, as a representation of the loss of electroconductivity on the heart *ex vivo*.^[^ ^35^ ^]^ Two isolated biceps femoris tissues placed 1 cm apart on the BCEP connect to the stimulator and sensors, respectively (Figure 6d). A pulsed DC waveform (2 V for 5 ms) applied to the left biceps femoris tissue passes to the right biceps femoris tissue within 1 ms, confirming that the BCEP can bridge and reconstruct the conductive pathways, as an essential feature in MI treatment.

## 3 Conclusion

The bioresorbable, elastic, and highly conductive cardiac patch described herein provides simple but useful functions as mechanical and electrical support layers for cardiac tissue regeneration. Compared with recently published studies (**Table** **1**), the BCEP has a significantly lower resistance and an acceptable modulus with unique bioresorbable properties. For example, phytic‐acid‐doped PANI on a chitosan substrate shows conductivity comparable to that of the BCEP but is relatively stiffer and non‐bioresorbable. The elastic moduli of hydrogel‐based cardiac patches are in the kilopascal range, but with low conductivity. Several commercialized patch products (e.g., PeriPatch by Neovasc Inc., Edwards bovine pericardial patch by Edwards Lifesciences, SJM Pericadial Patch with EnCap by St. Jude Medical, Inc.) are attractive for their nontoxicity and bioresorbability, but offer poor electroconductivity. The system reported here is a hybrid material structure, consisting of a serpentine structure of Mo as a bioresorbable metal integrated within a stretchable biodegradable POCO substrate. Additionally, porous POCO scaffolds can be prepared to enhance cell population for future cell delivery (Figure S10, Supporting Information). The device provides sufficient elastic behaviors for repeatable deformations to physiologically relevant strains (∼10%), to support the contractile function of cardiac tissue, where bioresorption occurs over a relevant time period. The BCEP is biocompatible and can promote maturation of hiPSC‐CMs. Synchronized contractions of the hiPSC‐CMs indicate that the Mo structure within the BCEP can effectively promote electrical signal propagation. *Ex vivo* experiments provide further validation of the promising electrical properties of the BCEP and of its potential use as a cardiac patch to improve heart function post‐MI.

**Table 1:** Summary of the characteristics of different cardiac patches

Polymer material	Conductive material	Resistance/conductivity	Modulus	Bioresorbable property	Ref
POCO	Mo	≈1.1 Ω	235 ± 50 kPa	Yes	This work
PLA	Au NW	67 kΩ	–	–	[9]
Chitosan scaffold	Phytic‐acid‐doped PANI	2.4 ± 0.9 × 10^−2^ S cm^−1^ (30–60 Ω cm^−1^)	6.73 ± 1.14 MPa	–	[19]
Chitosan film	Phytic‐acid‐doped PANI	10^−2^ S cm^−1^ (100 Ω cm^−1^)	0.06–1.53 ± 0.9 MPa	–	[36]
Gelatin/chitosan/PCL	–	–	300 kPa	Yes	[37]
PPy‐chitosan hydrogel	PPy‐chitosan hydrogel	25 × 10^−5^ S cm^−1^ (4 kΩ cm^−1^)	3 ± 0.5 kPa	Yes	[35]
Neonatal rat cardiomyocytes	Carbon nanotube	0.25–3.1 × 10^−3^ S (4–333 Ω)	–	–	[38]
Collagen	–	–	≈5 kPa (Elastic modulus)	Yes	[39]
Gelatin	Poly‐3‐amino‐4‐methoxybenzoic acid	20 × 10^−5^ S cm^−1^ (5 kΩ cm^−1^)	≈1 kPa (Young's modulus)	–	[40]

Note: poly(1,8‐octamethylene‐citrate‐co‐octanol) (POCO), polylactic acid (PLA), polyaniline (PANI), polypyrrole (PPy).

## 4 Experimental Section

### Preparation of the BCEP

The Mo serpentine structure was fabricated using a laser cutter (LPKF ProtoLaser R, LPKF Laser & Electronics). The POCO pre‐polymer was synthesized according to procedures based on a previous report.^[^ ^27^ ^]^ Briefly, 1,8‐ontandiol (Sigma–Aldrich), citrate acid (Sigma–Aldrich), and octanol (Acros Organics) were mixed and melted at 165 °C, followed by reaction at 140 °C for 3 h. The mixture was then dissolved in ethanol and precipitated in DI water for purification. The precipitate was collected and lyophilized for 24 h to obtain the final product. To fabricate the POCO patch, about 1 mL of the 40% POCO (w/w in ethanol) prepolymer solution was added to a glass slide (7.5 × 5 cm^2^) and left at room temperature overnight for solvent evaporation. Then, the slide was placed in an oven set to 80 °C for 4 days to cure the POCO patch. To fabricate the BCEP, the Mo serpentine structure was placed atop a viscous POCO sol after solvent evaporation and cured at 80 °C for 4 days.

### Mechanical Test

The mechanical properties of the samples were evaluated using an Instron universal testing machine (5944, Instron). To test Young's modulus, samples of dimensions 3 × 1.2 cm^2^ were stretched at a constant velocity of 15 mm min^−1^, and the force‐strain curves were recorded for calculation. To test the fatigue resistance, the BCEP was stretched for 1 00 000 cycles with 10% strain at 1 Hz to mimic the beating of a human heart. Square BCEPs (3 × 3 cm^2^) were fabricated for tensile tests along the longitudinal and transversal axes. The adhesion force between the Mo and POCO (samples of dimensions 5 × 1.2 cm^2^) was tested by peeling the two structures. The BCEP was adhered to rat skeletal muscle tissue and rat heart tissue upon UV irradiation for 10 min. After attachment, the heart tissue was flashed with DI water to show the adhesion between the BCEP and heart. In addition, the muscle strips adhered with the BCEP were severely deformed to show the robust adhesion between the BCEP and the muscle tissue. The two ends of the BCEP (≈0.5 × 0.5 cm^2^) were adhered to two tissues with separation of 1 cm. Then, the adhesion force was quantified by stretching of the two tissues with the Instron universal testing machine.

### Mechanical Simulation

3D finite element analysis (FEA) using the commercial software ABAQUS allowed study of the mechanical performance of the BCEP under stretching, bending, and twisting. Eight‐node 3D solid elements (C3D8R) were used for POCO and tissue, and four‐nodes shell elements (S4R) were used for the MO mesh layer. Convergence of meshes was tested to ensure computational accuracy. In the simulation, the Mooney–Rivlin strain energy potential model was used for the POCO (elastic modulus *E*_POCO_ = 260 kPa and Poisson's ratio *ν*_PU_ = 0.49) and tissue (elastic modulus *E*_tissue_ = 130 kPa and Poisson's ratio *ν*_tissue_ = 0.49) to display their hyperelastic material behaviors, where the relevant materials parameters are C10 = 0.0349 MPa, C01 = 0.0087 MPa, D1 = 0.4615 MPa^−1^ for POCO and C10 = 0.0174 MPa, C01 = 0.0043 MPa, D1 = 0.9230 MPa^−1^ for tissue. The elastic modulus and Poisson's ratio of Mo are *E*_Mo_ = 315 GPa, *ν*_Mo_ = 0.29, respectively. The elastic strain limit of Mo is ɛ_Mo elastic_ = 0.25%.

### Cell Culture

L929 fibroblasts were used to test the biocompatibility of the materials according to the ISO 10993 international standard. The cells were purchased from ATCC and cultured with DEME medium (4.5 g L^−1^ glucose with glutamine, Gioco) supplemented with 10% fetal bovine serum (FBS) and 1% penicillin‐streptomycin at 37 °C and 5% CO_2_. When the cells reached 80% confluency, they were seeded onto various samples at a concentration of 10 000 cells cm^−2^. After 3 days of culture, live/dead staining was performed using a cytotoxicity kit (Thermo Fisher) according to manufacturer instructions. Cell viability was tested using the alamarblue assay (Sigma). Briefly, 30 µM of resazurin was applied to the cells and incubated at 37 °C and 5% CO_2_ for 3 h. Then, about 100 µL of the solution from each sample was transferred to a 96‐well plate and fluorescence intensity was detected at a wavelength of 560/590 (excitation/emission). A blank sample (without cells) was used as the background control.

The hiPSC‐CMs were differentiated from hiPSCs according to a previous protocol.^[^ ^41^ ^]^ Briefly, 1.5 million hiPSCs were seeded into one well of a 12‐well plate. Fresh mTeSR medium (Stem Cell Technologies) was changed every day for 4 days. On the day of induction, the RPMI 1640 medium (Gibco) with 1x B‐27 minus insulin (Gibco) (RPMI/B27‐I) supplemented with 12 µM CHIR99021 (Selleckchem) was added to the cells. After 24 h of incubation, the old medium was replaced with the RPMI/B27‐I medium and cultured for an additional 48 h. On day 3, the RPMI/B27‐I medium supplemented with 5 µM IWP2 (Tocris) was added to the cells and incubated for 2 days. On day 5, the old medium was replaced with RPMI/B27‐I medium. On day 7, the RPMI 1640 medium with 1x serum‐free B‐27 (Gibco) (RPMI/B27) was added to the cells and changed every 3 days thereafter until use.

The hiPSC‐CMs were treated with 0.25% trypsin‐EDTA solution for 5 min at 37 °C and 5% CO_2_ to partially dissociate the cells (small cell aggregates can be observed). After centrifuging, the cells were resuspended with the RPMI 1640 medium supplemented with 20% FBS (RPMI‐20) and seeded onto the samples at a density of 20 000 cells cm^−2^ (based on single‐cell number counting). The Rock inhibitor Y27632 (Tocris) was used to promote initial cell adhesion and spreading on the samples. After 2 days of culture, the old medium was replaced with fresh RPMI‐20 medium and changed every other day. After an additional 5 days of culture (total 7 days for the patches), the hiPSC‐CMs were used for cardiac function analysis. For cell viability test, hiPSC‐CMs were seeded onto various samples as described above. After 3 days of culture cell viability was tested with the alamarblue assay.

### Immunofluorescence Staining

The hiPSC‐CMs were fixed with 4% paraformaldehyde and stained for specific cardiomyocyte markers, including α‐actinin and MLC‐2a. Briefly, the fixed cells were rinsed with PBS and treated with 0.1% triton X‐100 followed by 1% bovine serum albumin (BSA) blocking for 30 min. The primary antibodies of α‐actinin (Invitrogen, mouse‐anti‐human) and MLC‐2a (Invitrogen, rabbit‐anti‐human) were diluted at a ratio of 1:200 in 1% BSA and applied to the cells for overnight incubation at 4 °C. After 3 × 5 min rinsing with PBS, we applied DAPI, alexa fluor‐488 labeled goat antimouse secondary antibody, and alexa fluro‐594 labeled donkey antirabbit secondary antibody were applied to the cells and incubated at room temperature for 50 min. After 3 × 5 min PBS rinsing, the samples were imaged via a Cytation 5 cell imaging multimode reader (Bio Tek Instrument). The acquired images were analyzed using ImageJ software to compare the relative fluorescence intensities in accordance with the procedure in a previous report.^[^ ^42^ ^]^ Three independent samples were imaged with a 10x objective using a Cytation 5 Cell Imaging Multimode Reader. Three randomly selected regions were captured in each sample with the same exposure setup. Random selection should minimize artificial variations and accurately represent the cell population. The gap junction Cx43 was stained as described above without treatment with 0.1% triton X‐100.

### Flow Cytometry

After 7 days of culture the cells were detached from the substrates through trypsinization followed by fixation in 4% paraformaldehyde. Then the cells were probed forcompared α‐actinin and MLC‐2a, and Cx43 as described above for flow cytometry. An LSR Fortessa 2 Analyzer flow cytometer was used for the experiments. A total event of 10 000 was set as stop. The software FlowJo 10.8.1 was used for data analysis. For α‐actinin and MLC‐2a analysis, the diagram was divided as Q1 (α‐actinin+/MLC‐2a‐), Q2 (α‐actinin+/MLC‐2a+), Q3 (α‐actinin‐/MLC‐2a+), and Q4 (α‐actinin‐/MLC‐2a‐) regions based on fluorescence intensity (10^3^). The percentage of cells in each region was used to determine cell maturation. For Cx43 analysis, the histogram of Cx43 versus counts was plotted to calculate the percentage of positively stained cells.

### Assessment of hiPSC‐CM Contractions

Cytoplasmic Ca^2+^ was visualized using fluo‐4 AM (Invitrogen) to assess hiPSC‐CM contractions. After 7 days of culture of the samples, the cells were incubated with 5 µM fluo‐4 AM and incubated at 37 °C and 5% CO_2_ for 30 min, followed by rinsing with Tyrode's solution (Sigma). Then, the cells were imaged using the Cytation 5 imaging reader. Time‐lapse images were captured at 0.1 s intervals for 10 s. The converted video files were analyzed using MATLAB software.

### Animal Experiment

All animal studies were approved by IACUC at Northwestern University (IS00018748) following NIH guidance. Four skin capsules were created in each C57BL/6J mice (8w, female, Charles River) with implantation of POC, POCO, Mo, and BCEP patches. At 3‐days and 4‐weeks postsurgery, the animals were euthanized and the tissues were collected for histological staining according to previous publications to evaluate the biocompatibility of the different materials.^[^ ^43^ ^]^ Quantification of pan macrophage marker F4/80, M1 macrophage marker CD86, M2 macrophage marker CD163, and the thickness of fibrotic capsule were performed with the software ImageJ.

### Ex Vivo Assessment of Bioconductivity

All ex vivo studies were approved by the Institutional Animal Care and Use Committee (IACUC) at Northwestern University (protocol ISO00009470). Cardiac and muscle tissues were harvested from adult Sprague–Dawley rats. The fresh tissues were placed on the bioadhesive hydrogel‐coated BCEP and exposed to ultraviolet (UV) light for 5 min to crosslink the bioadhesive hydrogel. PowerLab 8/35 (PL3508, ADInstruments, US; differential inputs) was used to stimulate the tissues and measure the electrophysiological and voltage signals from the BCEP and tissues.

### Statistics

The data were analyzed using Kyplot software (version 2.0, KyensLab). A one‐way analysis of variance with Tukey's post‐hoc test was performed for multiple comparisons. An unpaired *t*‐test was used for comparison between the POCO and BCEP groups. The results are shown as mean ± SD, and a value of *p* ≤ 0.05 is considered to indicate a significant difference.

## Conflict of Interest

The authors declare no conflict of interest.

## Supporting information

Supporting Information

Supplemental Video 1

Supplemental Video 2

Supplemental Video 3

## Data Availability

The data that support the findings of this study are available from the corresponding author upon reasonable request.
