# Supplementary material for: Materials and Design Approaches for a Fully Bioresorbable, Electrically Conductive and Mechanically Compliant Cardiac Patch Technology
Source: Adv Sci (Weinh). 2023 Jul 30;10(27):2303429. doi: 10.1002/advs.202303429 (PMC10520666; doi:10.1002/advs.202303429)
Supplement: Supplementary file 1 — Supporting Information [file ADVS-10-2303429-s001.pdf]

ADVANCED SCIENCE
Open Access

Supporting Information

for *Adv. Sci.*, DOI 10.1002/advs.202303429

Materials and Design Approaches for a Fully Bioresorbable, Electrically Conductive and Mechanically Compliant Cardiac Patch Technology

Hanjun Ryu, Xinlong Wang, Zhaoqian Xie, Jihye Kim, Yugang Liu, Wubin Bai, Zhen Song, Joseph W. Song, Zichen Zhao, Joohee Kim, Quansan Yang, Janice Jie Xie, Rebecca Keate, Huifeng Wang, Yonggang Huang, Igor R. Efimov, Guillermo Antonio Ameer* and John A. Rogers**

Supporting Information

Materials and design approaches for a fully bioresorbable, electrically conductive and mechanically compliant cardiac patch technology

Hanjun Ryu, Xinlong Wang, Zhaoqian Xie, Jihye Kim, Yugang Liu, Wubin Bai, Zhen Song, Joseph W. Song, Zichen Zhao, Joohee Kim, Quansan Yang, Janice Jie Xie, Rebecca Keate, Huifeng Wang, Yonggang Huang, Igor R. Efimov, Guillermo Antonio Ameer*, John A. Rogers**

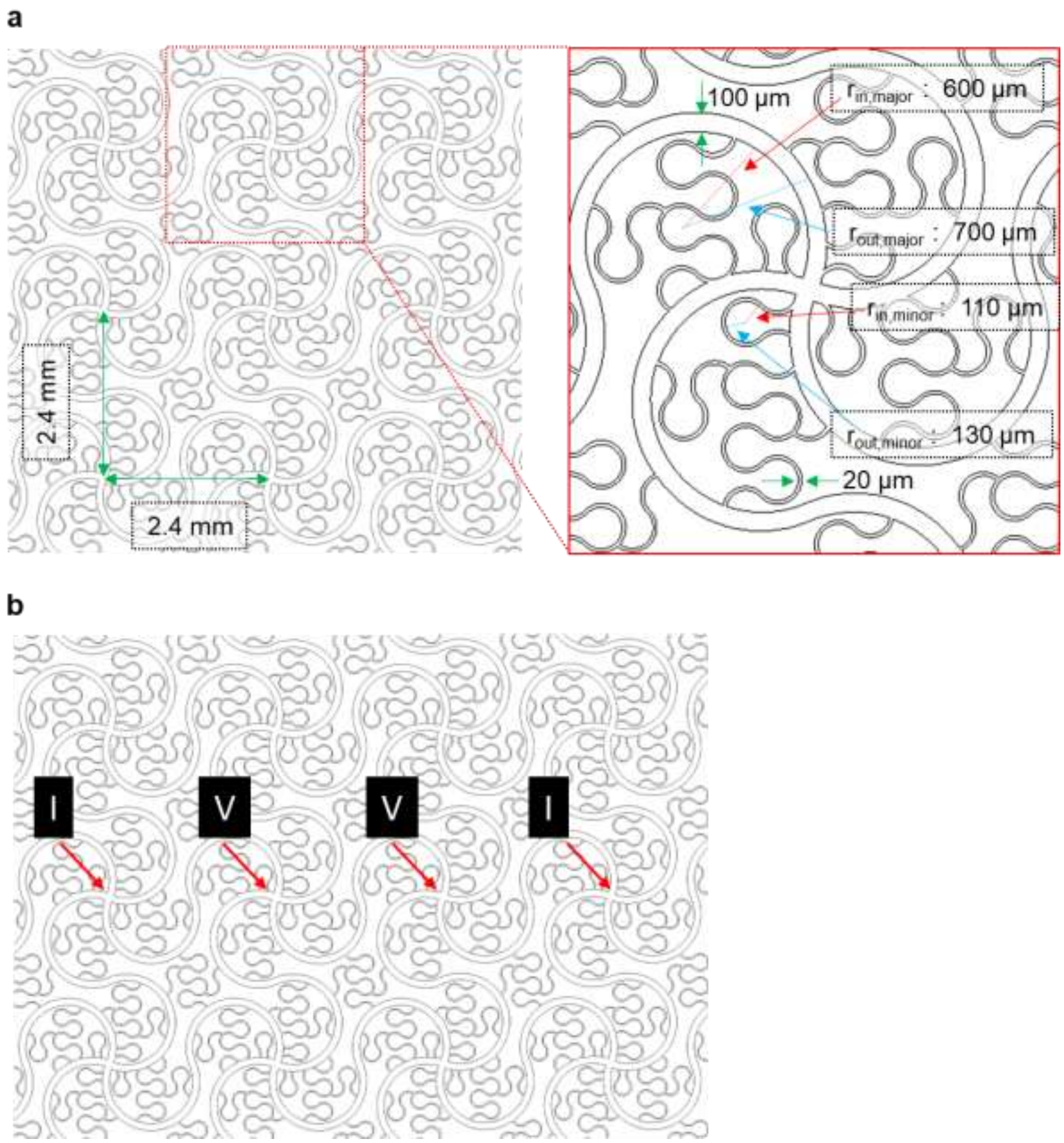

Figure S1. (a) Design of the Mo mesh, with an inset image that provides a magnified view. (b) positions of probes for four-point electrical evaluations of the Mo mesh.

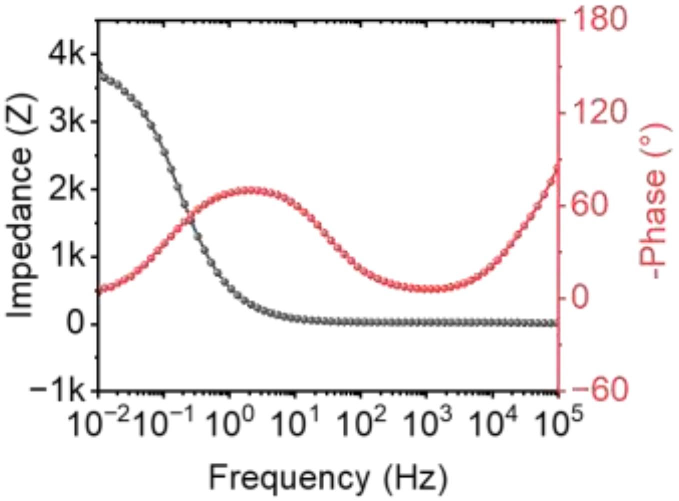

Figure S2. Representative impedance and phase plots of the interface between the BCEP and DPBS as functions of frequency.

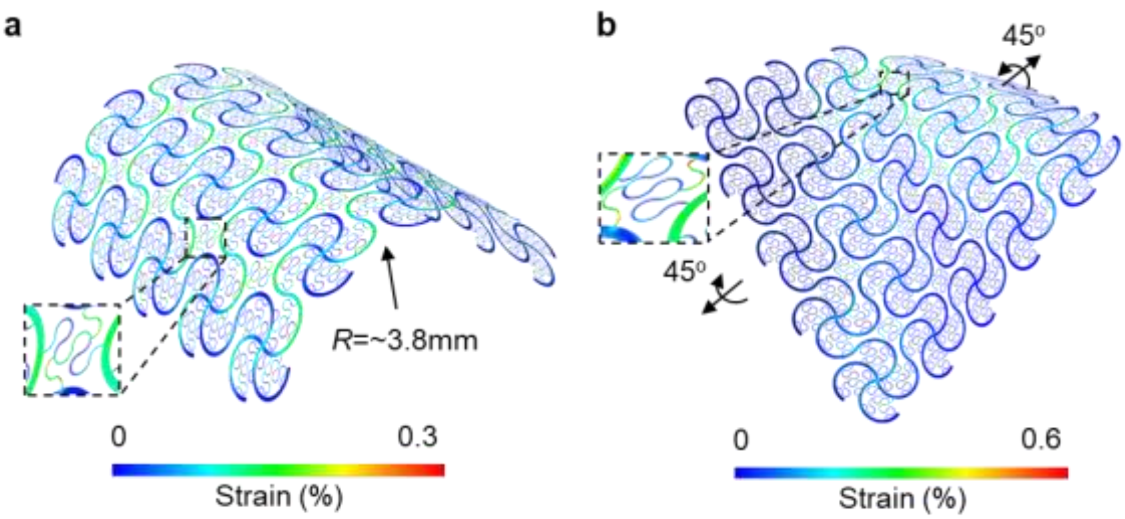

Figure S3. Finite element analysis results of the strain distribution in the Mo mesh layer during (a) bending and (b) twisting. The colors represent the equivalent strain.

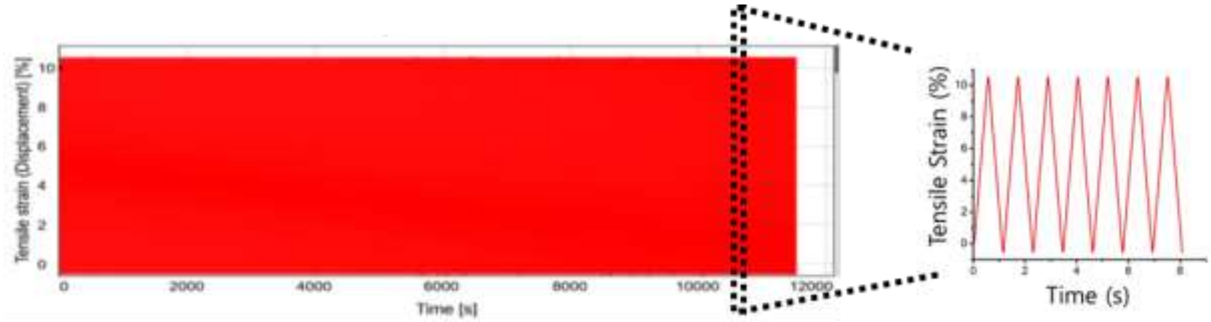

Figure S4. Cyclic test results of the BCEP for 10,000 iterations.

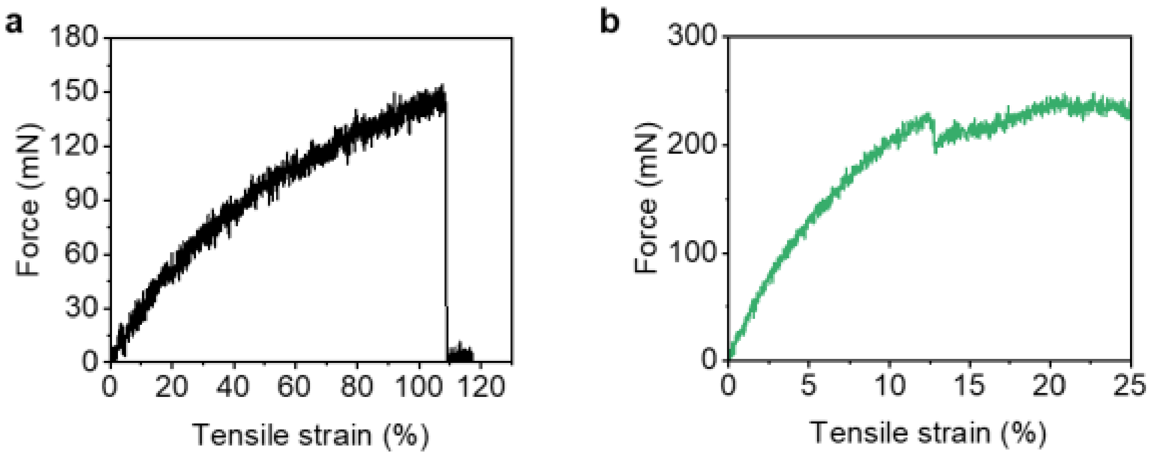

Figure S5. Force as a function of uniaxial tensile strain for (a) bioadhesive hydrogel and (b) BCEP with a coating of bioadhesive hydrogel.

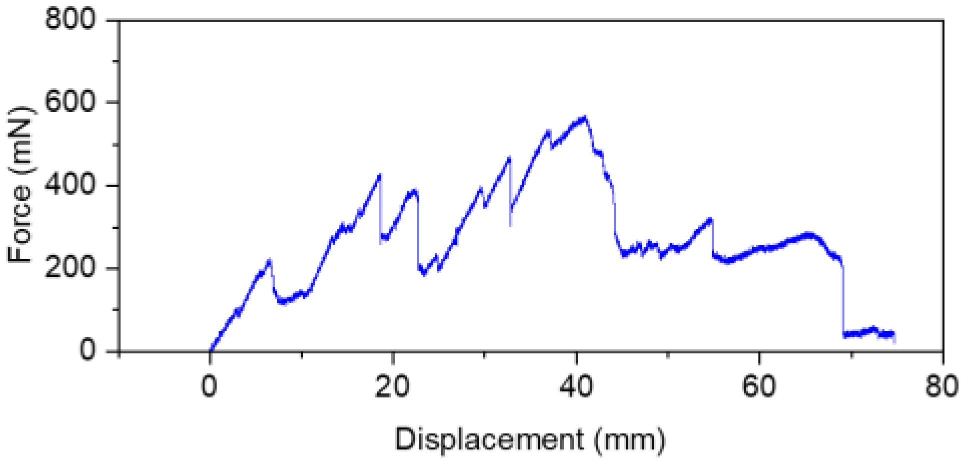

Figure S6. Applied force as a function of tensile displacement during peel testing of a sample of Mo mesh and POCO after immersion in DPBS for ~24 hours.

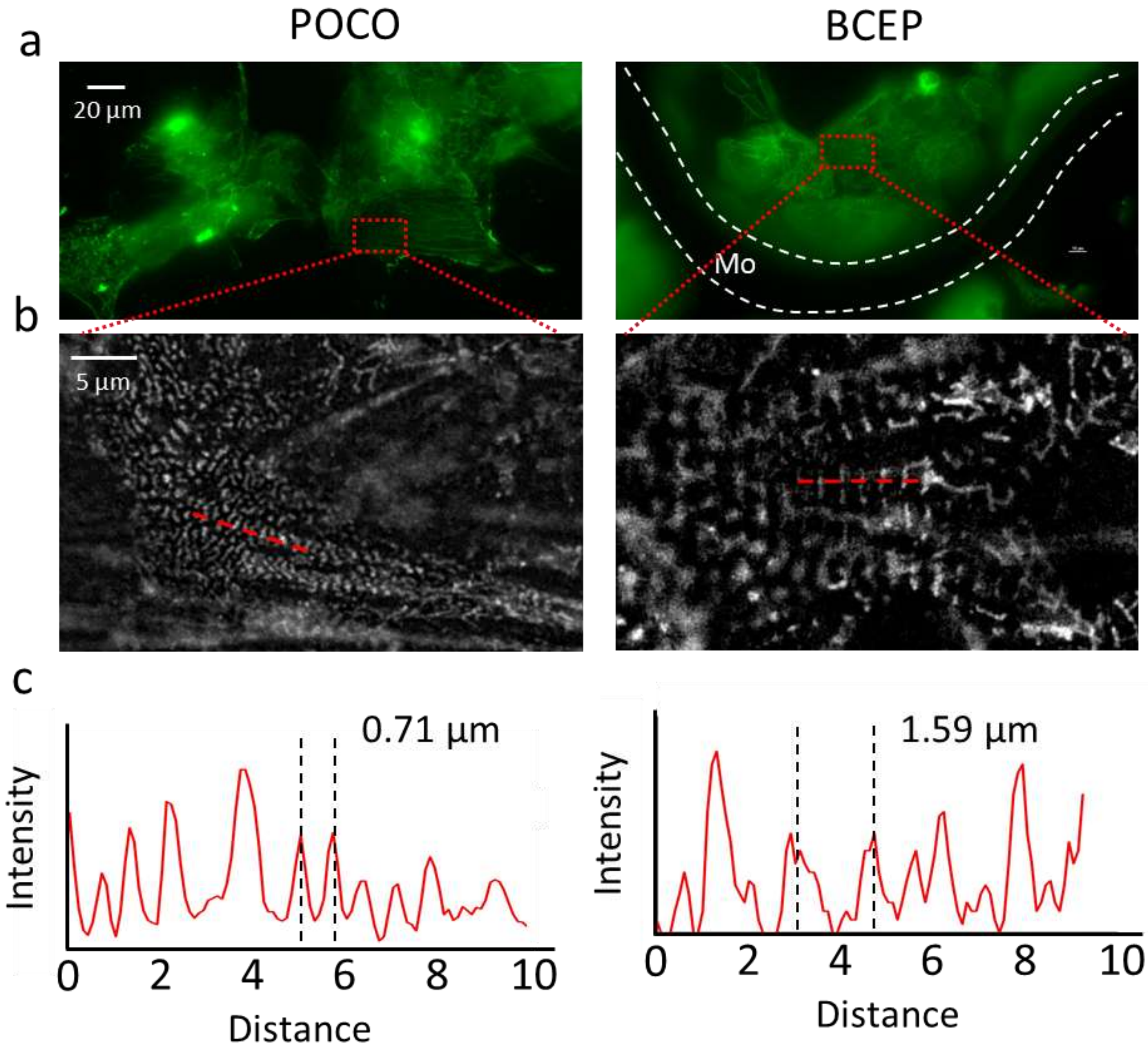

Figure S7. (a) Probing of α-actinin in hiPSC-CMs on POCO and BCEP patch. (b) High magnification image of the square zone in (a). (c) intensity plot along the red line in (b).

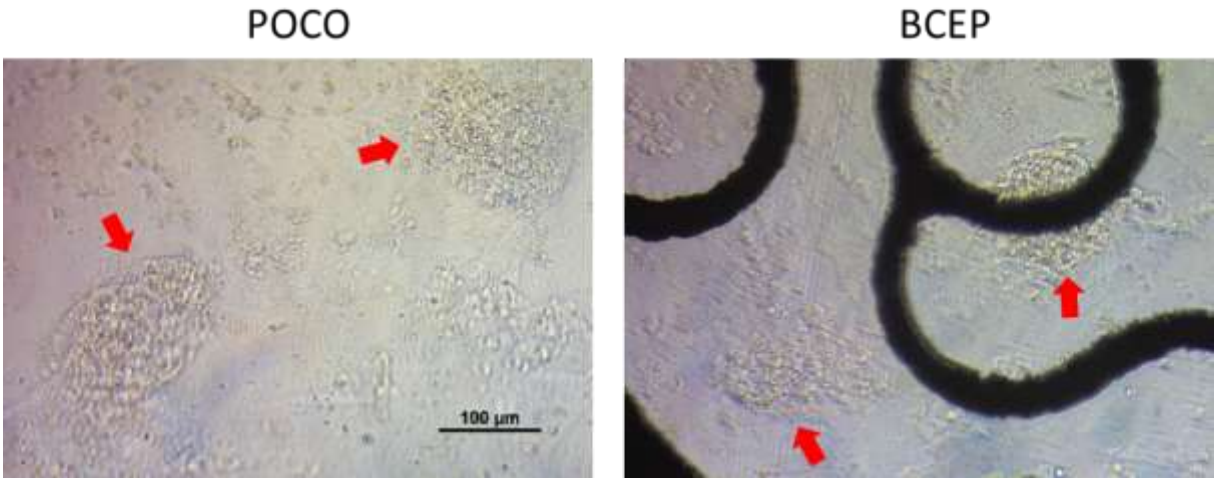

Figure S8. Phase contrast images showing isolated hiPSC-CM clusters on POCO and BCEP. The red arrows indicate cell clusters, and the dark serpentine structure is the Mo mesh.

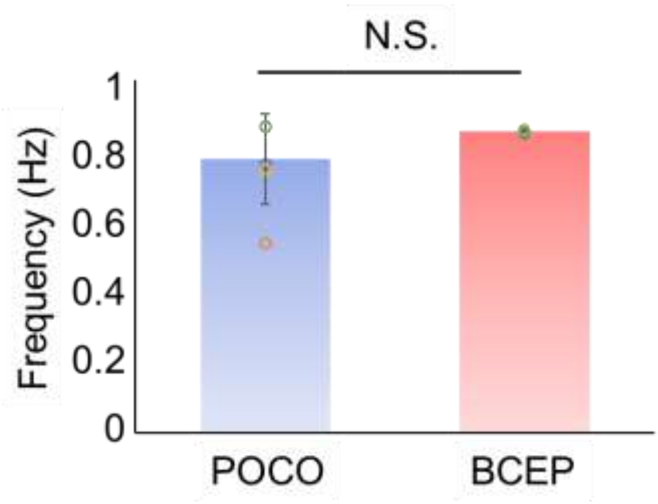

Figure S9. Cell cluster beating frequency on POCO and BCEP patches. n=6, N.S., no significant difference.

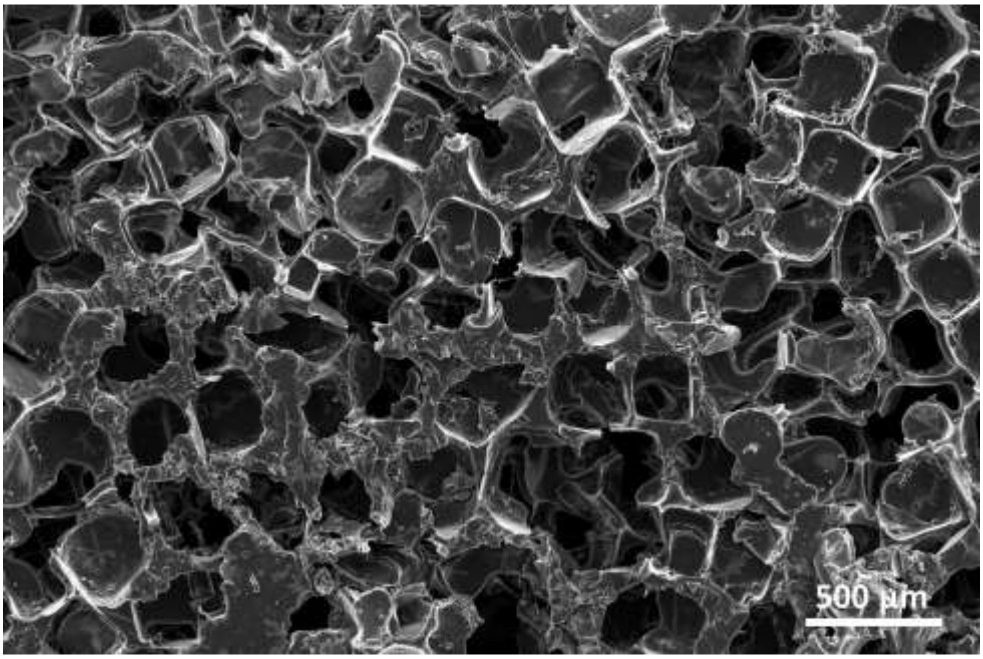

Figure S10. SEM image of porous POCO scaffold.

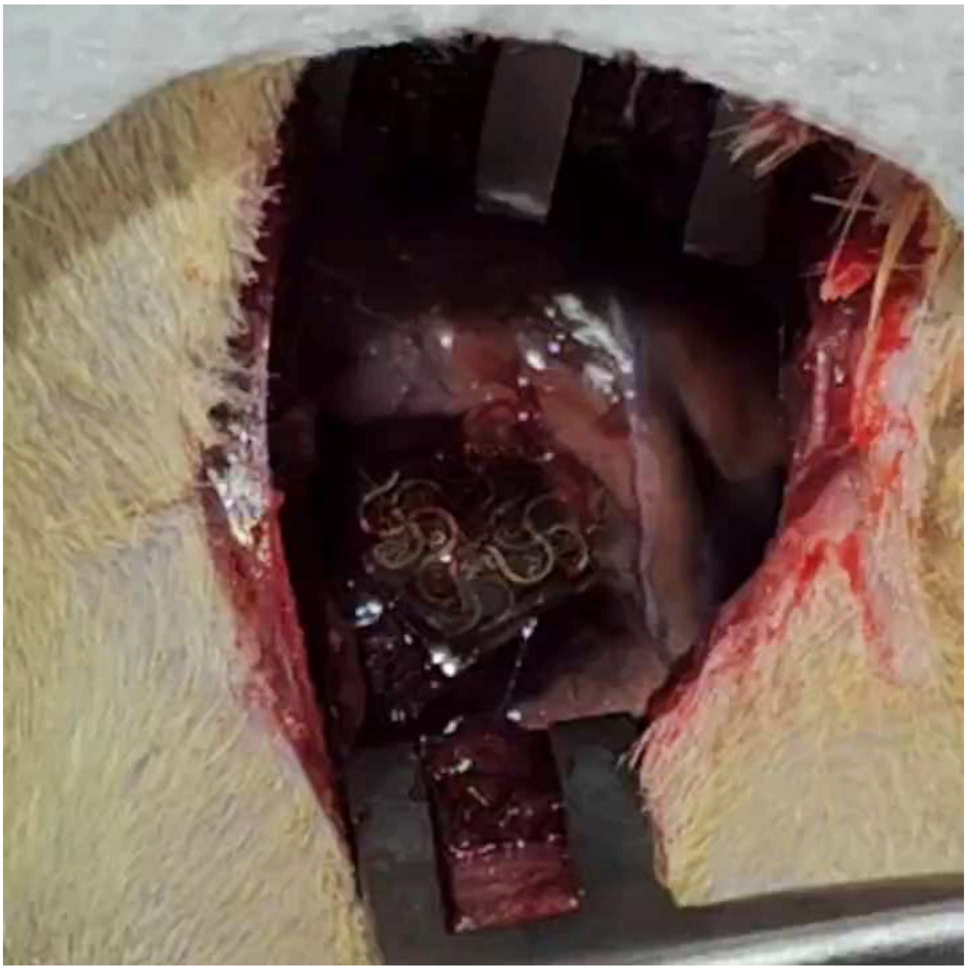

Video S1. Application of BCEP on a rat heart.

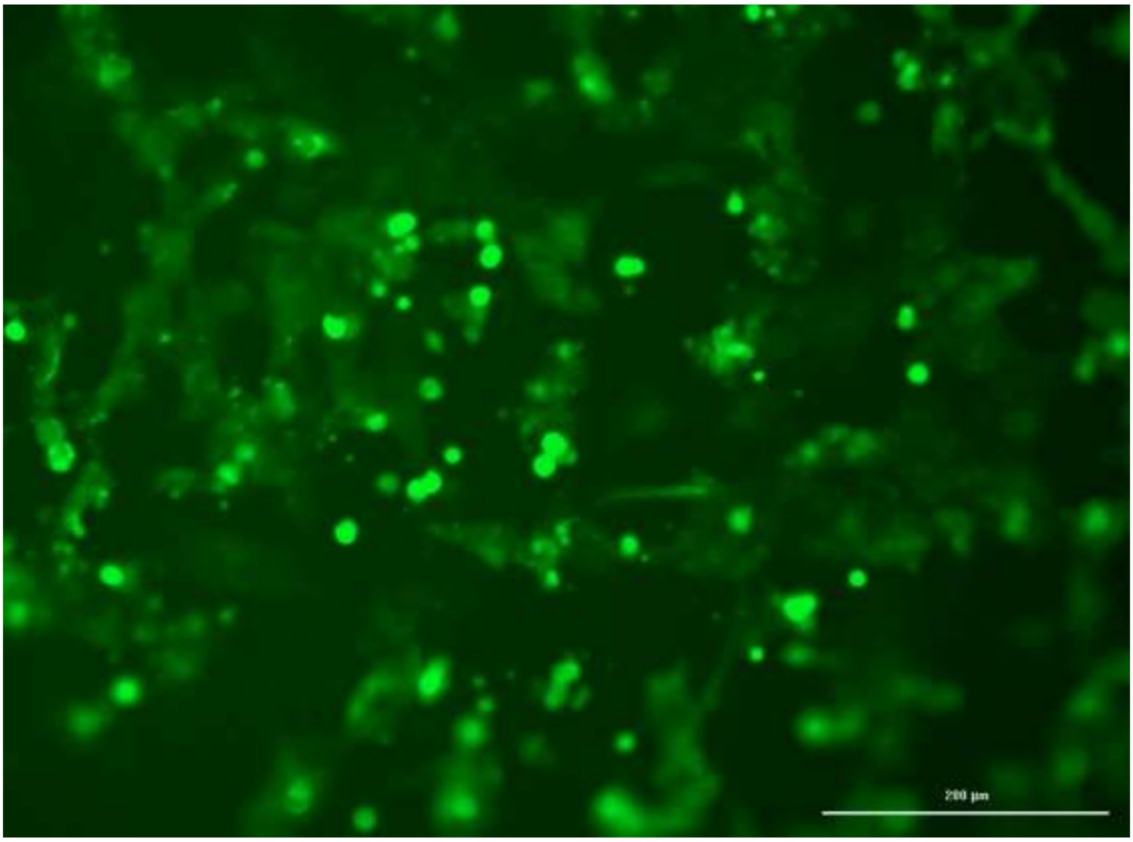

Video S2. hiPSC-CMs contraction on POCO patch.

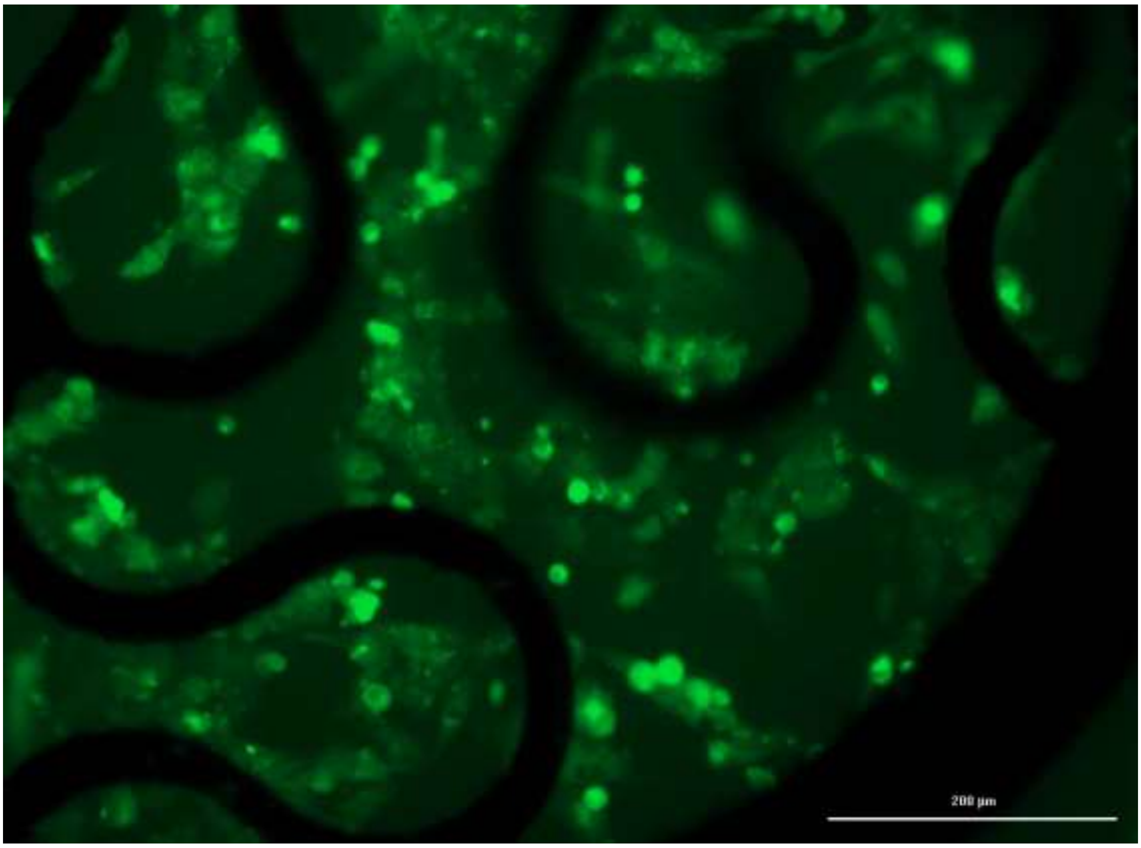

Video S3. hiPSC-CMs contraction on BCEP.
